# Internal and external heat load with fire fighter protective clothing: data from the lab and the field

**DOI:** 10.1186/2046-7648-4-S1-A100

**Published:** 2015-09-14

**Authors:** Simon Annaheim, Fabio Saiani, Marc Grütter, Piero Fontana, Martin Camenzind, Rene Rossi

**Affiliations:** 1 Laboratory for Protection and Physiology, Empa, St. Gallen, Switzerland

## Introduction

Protective clothing for fire fighters is characterized by high thermal insulation to reduce external heat gain through dry and wet heat [1,2]. However, these protective properties limit heat dissipation from the human body and impede thermoregulation [3,4]. As a result, fire fighters suffer from non-compensable heat stress during physical activity especially when exposed to a harsh environment [5]. However, there is little data available about the effect of internal and external heat load on whole-body thermo-physiological responses when wearing protective clothing. This study provides data of a lab trial including physical activity and a field test conducted in a fire chamber.

## Methods

The lab trial (climatic chamber, n = 10 participants) included two exposures to 40 °C ambient temperature including physical activity (treadmill with 3.3 and 5 Mets) of 20 min duration with a recovery phase of 20 min duration at 25 °C in between. The field trial (fire chamber, n = 9 participants) consisted of an exposure to a mean (SD) temperature at 1m above ground level of 141(18) °C for 15 min without any kind of activity. As for the lab trial, two exposures were separated by a recovery phase of 15 min duration at 25 °C. In both investigations, rectal temperature (T_re_), heart rate (HR) and sweat water loss (change in body mass) were measured. Mean skin temperature (${\bar{\text{T}}}_{\text{sk}}$) was calculated according to EN ISO 9886 based on temperature measures at 8 body sites [6].

## Results

In the lab and field trials respectively, similar changes in rectal temperature were observed for the first (+0.15(0.05) °C.10 min^-1 ^and +0.17(0.11) °C.10 min^-1^) and the second exposure (+0.28(0.07) °C.10 min^-1 ^and +0.29(0.13) °C.10 min^-1^). The maximum values for both trials are shown in table 1.

**Table 1:** Mean (SD) maximum values observed during lab and field tests.

	T_re _[°C]	${\bar{\text{T}}}_{\text{sk}}$ [°C]	HR [bpm]	Sweat loss [kg]
Climatic chamber trial	38.1(0.3)	37.0(0.2)	139.1(17.3)	0.9(0.2)

Fire chamber trial	38.6(0.3)**	41.8(1.0)***	164.6(13.7)**	1.2(0.3)*

* *P *< 0.05, ** *P *< 0.01. *** *P *< 0.001

## Discussion

Maximum physiological responses during the fire chamber trial were more pronounced as for the climatic chamber trial. This observation underlines the importance of fire fighter protective clothing in harsh conditions. However, changes in thermo-physiological responses were found to be similar for the lab and the field trial.

## Conclusions

Our findings highlight the importance of the thermo-physiological impact of protective clothing during activity. Increased physical activity itself can lead to non-compensable heat stress when wearing protective clothing, even though the environmental condition is not expected to induce severe heat stress.

## References

[B1] Rossi R Hot steam transfer through heat protective clothing layers Int J Occup Saf Ergon 2004 10 3 239 45 15377408 10.1080/10803548.2004.11076611

[B2] Morel A A review of heat transfer phenomena and the impact of moisture on firefighters' clothing and protection Ergonomics 2014 57 7 1 12 24734933 10.1080/00140139.2014.907447

[B3] Cheung SS The thermophysiology of uncompensable heat stress. Physiological manipulations and individual characteristics Sports Med 2000 29 5 329 59 10.2165/00007256-200029050-00004 10840867

[B4] Holmér I Protective clothing in hot environments Ind Health 2006 44 3 404 413 10.2486/indhealth.44.404 16922184

[B5] Cheung SS Physiological strain and countermeasures with firefighting Scand J Med Sci Sports 2010 20 3 103 16 21029197 10.1111/j.1600-0838.2010.01215.x

[B6] EN ISO 9886 Ergonomics - Evaluation of thermal strain by physiological measurements 2004

